# *Bacillus anthracis* co-opts nitric oxide and host serum albumin for pathogenicity in hypoxic conditions

**DOI:** 10.3389/fcimb.2013.00016

**Published:** 2013-05-17

**Authors:** Stephen St. John, Ryan Blower, Taissia G. Popova, Aarthi Narayanan, Myung-Chul Chung, Charles L. Bailey, Serguei G. Popov

**Affiliations:** National Center for Biodefense and Infectious Diseases, School of Systems Biology, George Mason UniversityManassas, VA, USA

**Keywords:** anthrax, toxicity, microaerobic culture, nitric oxide, nitric oxide synthase

## Abstract

*Bacillus anthracis* is a dangerous pathogen of humans and many animal species. Its virulence has been mainly attributed to the production of Lethal and Edema toxins as well as the antiphagocytic capsule. Recent data indicate that the nitric oxide (NO) synthase (baNOS) plays an important pathogenic role at the early stage of disease by protecting bacteria from the host reactive species and S-nytrosylating the mitochondrial proteins in macrophages. In this study we for the first time present evidence that bacteria-derived NO participates in the generation of highly reactive oxidizing species which could be abolished by the NOS inhibitor L - NAME, free thiols, and superoxide dismutase but not catalase. The formation of toxicants is likely a result of the simultaneous formation of NO and superoxide leading to a labile peroxynitrite and its stable decomposition product, nitrogen dioxide. The toxicity of bacteria could be potentiated in the presence of bovine serum albumin. This effect is consistent with the property of serum albumin to serves as a trap of a volatile NO accelerating its reactions. Our data suggest that during infection in the hypoxic environment of pre-mortal host the accumulated NO is expected to have a broad toxic impact on host cell functions.

## Introduction

*Bacillus anthracis* is the causative agent of anthrax. Although the incidence of disease among people in the developed countries is low, it remains important as a biodefense threat. Antibiotics are the only approved drugs for anthrax treatment, which is effective only at the early stages of infection. Patients with the advanced disease have about 50% chance of survival (Inglesby et al., 2002). Therefore, further understanding of *B. anthracis* toxicity is required for the acceleration of progress in the development of novel anthrax therapies and prophylaxes.

The disease can be initiated by three major routes: inhalation, ingestion of spores, as well as a direct contact of spores with damaged skin (Inglesby, 2002). During inhalational anthrax, *B. anthracis* spores are internalized by resident phagocytes (alveolar macrophages or dendritic cells) and transported to the regional lymph nodes (Dixon et al., 2000; Guidi-Rontani, 2002). Inside macrophages, some internalized spores survive a bactericidal environment and ultimately initiate disease by escaping the macrophages (Cote et al., 2008). The spores also demonstrate a capacity of invading the lung epithelium directly at low frequency (Russell et al., 2008). During vegetative growth, bacterium produces several virulence factors including the toxins, such as the Lethal Toxin (LT) and Edema Toxin (ET), and a poly-γ-D-glutamic acid capsule [reviewed in Moayeri and Leppla (2009) and Guichard et al. (2012)]. LT and ET consist of the receptor-binding protective antigen (PA) associated with the catalytic subunits, Lethal Factor and Edema Factor, respectively. The toxins' genes are expressed from plasmid XO1, while the capsule gene is located on the plasmid XO2. In macrophages, LT causes intracellular proteolytic cleavage of members of the mitogen-activated protein kinase kinase (MAPKK) family. ET is a calcium- and calmodulin-dependent adenylyl cyclase that converts cytosolic ATP to cAMP (Moayeri and Leppla, 2009). Accumulated evidence demonstrates that LT and ET influence many important cellular processes including the host's innate immune response; however, mechanisms by which *B. anthracis* kills the host are not fully understood.

Recent data obtained in animal models of anthrax using the virulent strains with deletions of LT and ET genes show that *B. anthracis* possesses pathogenic factors which can surpass the effects of these toxins (Heninger et al., 2006; Chand et al., 2009; Levy et al., 2012a,b; Lovchik et al., 2012). For example, Heninger et al. (2006) demonstrate that LT and ET are not required for a full toxicity of Ames strain upon an inhalation administration of spores. However, these studies provided no mechanistic interpretation of their results.

We have been interested in investigation of the pathogenic mechanisms contributing to the LT-independent virulence with a particular focus on the contribution of *B. anthracis* nitric oxide (NO) synthase (baNOS). Similar to mammalian NOSs, the bacterial homolog generates NO from L-arginine in the presence of oxygen (Sudhamsu and Crane, 2009; Crane et al., 2010). NO is a relatively unreactive free radical. Easy diffusion of NO through membranes (Denicola et al., 1996b) makes possible its interactions with intracellular targets. In the host cells, NO and other reactive nitrogen species (RNS) derived from NO participate in numerous biological events such as glycolysis, growth, signal transduction, stress response and maintenance of homeostasis by S-nitrosylation of protein thiol groups and nitration of tyrosine residues (Habib and Ali, 2011). S-nitrosylation is a ubiquitous posttranslational, enzyme-independent, redox-sensitive modification that serves as a major effector of NO-mediated biochemistry regulating broad spectrum of proteins. NO can also react with superoxide (O^·−^_2_) and form highly-toxic peroxynitrite (ONOO^−^) playing an important role in different inflammatory diseases. Peroxynitrite is formed during sepsis, inflammation, excitotoxicity, and ischemia-reperfusion of tissues, conditions under which the cellular production of NO and superoxide increase (Pacher et al., 2007), and participates in reactions related with the pathological expression of these processes. Peroxynitrite induces nitration of protein tyrosine residues (3-nitrotyrosine) resulting in modulation of catalytic activity, cell signaling, and cytoskeletal re-organization (Pacher et al., 2007).

Available data indicate that baNOS plays an essential role in *B. anthracis* virulence through different mechanisms relevant to the early stage of disease involving interaction of bacteria with macrophages as well as the late, pre-mortal stage characterized by the interaction of pathogenic factors with non-phagocytic host cells. One of these mechanisms confers protection of *B. anthracis* from the host reactive species within macrophages by preventing DNA damage during the Fenton reaction of ferrous ion with hydrogen peroxide (Shatalin et al., 2008). We recently reported that S-nitrosylation of mitochondrial proteins by baNOS-derived NO leads to depletion of the macrophage bioenergetics resulting in cell death (Chung et al., 2013). In agreement with these observations, the *B. anthracis* mutant with a deletion of baNOS gene is strongly attenuated in mice (Shatalin et al., 2008).

Another LT-independent mechanism likely relevant to late-stage anthrax was demonstrated in experiments with non-phagocytic host cells exposed to pathogenic factors generated by *B. anthracis* Sterne and dSterne strains grown in microaerobic conditions (Popova et al., 2011). The Sterne strain is fully toxigenic but attenuated due to the absence of capsule. The dSterne strain is a derivative of Sterne producing neither LT, ET nor the capsule. Compared to the fully aerobic cultures, the oxygen pressure in microaerobic cultures is reduced, but not completely abated, while the pressure of carbon dioxide is increased. It was found that the intoxication depends on the expression of the pore-forming hemolysin, anthrolysin O (ALO). The killing activity of ALO is synergistically enhanced by the bacterial metabolite, succinic acid (SA), released from bacteria into environment as a result of anaerobic fermentation. Host cells exposed to bacterial culture supernatants (Sups), demonstrate the onset of acute oxidative stress, which can be attenuated by the mimetics of superoxide dismutase (SOD) and catalase, indicating the involvement of the superoxide radical or other reactive oxygen species (ROS). As a substrate of the mitochondrial complex II, SA can stimulate generation of ROS by the host cells in hypoxic conditions (Quinlan et al., 2012). The toxic role of ALO in this process likely consists in perforating the cytoplasmic membrane and creating pores for the delivery of SA and other bacterial products into the target cells (Popova et al., 2011).

However, the ALO-based mechanism contributes only to about a half of the Sups' toxicity as it follows from the effect of ALO inhibition by cholesterol (Popova et al., 2011). The partial protective effect of the iron porphyirin derivative, FeTPPS, used in previous studies to catalyze decomposition of peroxynitrite (Belik et al., 2010; Valez et al., 2012) indicates that this RNS may also play role in the toxicity of Sups. Due to the transient nature of peroxynitrite, it is difficult to observe it directly within the cells. In the conditions of oxidative stress, this unstable cytotoxic compound can be formed in the fast reaction of superoxide with NO derived from the host NOS or baNOS (Pacher et al., 2007). Another source of NO may consist in denitrification of nitrate or nitrite in the conditions of anaerobic respiration demonstrated for a number of bacterial species (Kraft et al., 2011; Bueno et al., 2012).

In this follow-up study, we thought to characterize the nature of cytotoxic species formed in Sups during a microaerobic growth of *B. anthracis*. We present evidence consistent with the notion that NO produced by baNOS can participate in the generation of *B. anthracis* toxicity to non-phagocytic cells through formation of peroxynitrite and its reaction products. During experimentation with culture media used for bacterial growth we found that the presence of bovine serum albumin (BSA) potentiated the toxicity of bacteria, and explored the mechanistic features of this phenomenon. The results indicate that BSA serves as a trap capturing and stabilizing a volatile NO produced by *B. anthracis*. We conclude that the role of NO in anthrax is not limited to its low-level production within macrophages. During infection the bacteria are likely to accumulate NO in circulation due to the high content of albumin in serum. In a hypoxic environment of the pre-mortal host accompanied by the presence of ROS the accumulated NO is expected to have a broad systemic impact on different cell types through cytotoxicity of its downstream products and protein chemical modifications causing deterioration of host cell functions.

## Materials and methods

### Reagents

All reagents including superoxide dismutase from *E. coli*, peroxidase from horseradish, catalase from bovine liver, rabbit anti-nitrotyrosine antibody, and 5,5′-dithiobis-2-nitrobenzoic acid (DTNB) were from Sigma-Aldrich. BSA was of >98% purity, essentially free from fatty acids and globulin. Amplex Red (AR) and Alamar Blue dyes were from Invitrogen. Anti-rabbit IgG, HRP-linked antibody was from Cell Signaling Technology. All cell culture reagents and formulated media were purchased from Mediatech, Inc., VA. Complete Serum-Free Medium® (CSFM) is a proprietary serum-free and low-protein formulation based on DMEM/F12, RPMI 1640, and McCoy's 5A. It does not contain any insulin, transferrin, cholesterol, growth or attachment factors. The manufacturer indicates that the medium contains trace elements, high-molecular-weight carbohydrates, extra vitamins, a high-quality bovine serum albumin (1 g/l). Our analysis shows that it contains *ca.* 300 μM nitrate.

### Cell culture and bacterial strains

Primary human small-airway lung epithelial cells (HSAECs) were from Cambrex, Inc., MD. Cells were seeded at density 2.5 × 10^4^/well and grown till confluence in DMEM/F12 medium supplemented with non-essential amino acids, pyruvate, β-mercaptoethanol and 10% fetal calf serum at 37°C, 5% CO_2_ using 96-well culture plates. In challenge experiments the growth medium was removed, the cells were washed three times with warm HEPES-buffered saline (HBSS) and then incubated at 37°C, 5% CO_2_ without shaking with 200 μl/well of Sups for 2 h, unless specified otherwise. Viability of cells after challenge was routinely determined using the redox dye Alamar Blue which is a water-soluble, non-toxic, fluorometric/colorimetric growth indicator. Cellular growth and metabolism reduce the dye and change its color and fluorescence. Briefly, the cells were washed with HBSS, 100 μl of Alamar Blue in CSFM were added, incubated with cells until the accumulation of sufficient signal (typically from 30 min to 1 h), and fluorescence was measured at 530/584 nm. The metabolic activity of treated cells was calculated relative to mock controls.

Sups were produced by inoculation of spores (final 6× 10^6^ spores/ml) into the DMEM/F12 culture medium supplemented with non-essential amino acids, piruvate, glutamine, BSA, and 5 μM nitrate (complete DMEM/F12), unless indicated otherwise. The cultures were incubated at 37°C, 5% CO_2_ in 12-well tissue culture plates in static conditions or with shaking for 24 h. Bacterial growth was measured by optical density at 600 nm (OD_600_) using 96-well plates and 200 μl of suspension per well. Bacteria were removed by centrifugation, and Sups were supplemented with 100 μg/ml of streptomycin and 100 U/ml of penicillin to exclude growth of any contaminating bacteria.

The Sterne strain 34F2 (pXO1^+^, pXO2^−^) was obtained from the Colorado Serum Co. (Boulder, CO). The generation and characterization of the plasmidless delta Sterne strain were described in (Bradburne et al., 2008). The spores of *B. anthracis* strains were prepared as described (Popov et al., 2002).

In the experiments with L-NAME, different concentrations of the inhibitor were made by mixing 1 M stock solution in water with a complete DMEM/F12 buffered with 50 mM HEPES to maintain a uniform pH of 6.95–7.0 of all mixtures.

### Reverse transcriptase-polymerase chain reaction (RT-PCR)

Bacteria were cultured in DMEM containing 10% fetal calf serum and 2 mM glutamine at 37°C, 5% CO_2_ incubator without agitation or with agitation at 200 rpm. Trizol solution with bacterial enhancement reagent (Invitrogen) was used to isolate total RNAs from *B. anthracis*. Random primed cDNA was prepared from 1 μg of total bacterial RNA using Superscript II reverse transcriptase (Invitrogen). Semi-quantitative PCR of the cDNA was performed using Platinum Supermix (Invitrogen) and primers specific for bNOS [5′CTT GTC TTT CCA TAA TGT ACC (sense) and 5′TAA ATA TGC AAC GAA CGA CG (antisense)] to yield a 540-bp amplicon.

### Mass spectrometry

The Sups were dried with SpeedVac, reconstituted in 8 M urea, reduced by 10 mM DTT for 30 min, alkylated by 50 mM iodoacetamide for 30 min, and digested by trypsin at 37°C overnight. Tryptic peptides were further purified by Zip-Tip (Millipore) and analyzed by LC-MS/MS using a linear ion-trap mass spectrometer (LTQ, Orbitrap). After sample injection, the column was washed for 5 min with mobile phase A (0.4% acetic acid) and peptides eluted using a linear gradient of 0% mobile phase B (0.4% acetic acid, 80% acetonitrile) to 50% mobile phase B in 30 min at 250 nl/min, then to 100% mobile phase B for an additional 5 min. The LTQ mass spectrometer was operated in a data-dependent mode in which each full MS scan was followed by five MS/MS scans where the five most abundant molecular ions were dynamically selected for collision-induced dissociation using a normalized collision energy of 35%. Tandem mass spectra were searched against SEQUEST database using tryptic cleavage constraints. High-confidence peptide identifications were obtained by applying the following filters to the search results: cross-correlation score 1.9 for 1+, 2.2 for 2+, 3.5 for 3+ ions, and a maximum probability for a random identification of 0.01.

### AR assay

The enzymatic determination of hydrogen peroxide can be accomplished with high sensitivity and specificity using Amplex® Red (N-acetyl-3,7-dihydroxyphenoxazine, AR), a highly sensitive and chemically stable fluorogenic probe. Enzyme-catalyzed oxidation of AR, which is a colorless and non-fluorescent derivative of dihydroresorufin, produces highly fluorescent resorufin, which is detected by absorbance at 571 nm.

The experiments with AR measured the accumulation of resorufin in bacterial cultures grown as described above. AR and horseradish peroxidase (HRP) were added to culture medium prior to incubation at final concentrations of 0.1 mM and 0.2 U/ml, respectively. Samples of cultures were taken at specific time points, the bacteria were pelleted by centrifugation at 10,000 g for 5 min, and absorbance of Sups was read at 571 nm. The results in particular cultivation conditions demonstrated a satisfactory reproducibility between independent experiments. However, we detected some variation in the shape of the accumulation curves depending on the culture plate well size, volume and nature of medium in the wells likely caused by disturbance in the gas-liquid exchange of the volatile reaction products such as NO, NO_2_ upon handling of the microaeroibic cultures.

### Preparation of modified BSA

Sodium nitrite (400 mg/ml) was acidified to pH 5 using 0.5 M HCl, and mixed with equal volume of BSA solution (200 mg/ml in water). The mixture was incubated for 1 h at room temperature in the dark, and the protein was precipitated with four volumes of ice-cold acetone. After incubating at minus 20°C for 20 min the protein was pelleted, washed 4× with 70% acetone, air dried and resuspended in 100 mM HEPES, 1 mM EDTA, 0.1 mM neocuproine, 1% Tween 20, pH 7.5.

The SH content of BSA was determined using a thiol-specific Ellman's reagent (DTNB) as described by the manufacturer.

### Statistical analyses

All measurements were made in triplicates, and all experiments were repeated at least twice with consistent results. Error bars in the figures indicate standard deviations (*n* = 3) or 95% confidence intervals (two-tail *t*-test).

## Results

### BSA increases accumulation of no reaction products and the toxicity of Sups

**Figure 1:**
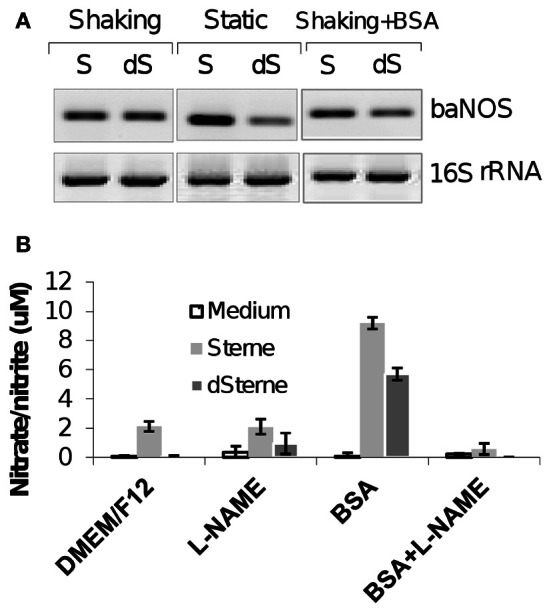
**BaNOS is expressed in aerated and static cultures, which accumulate nitrate/nitrite as trace products of NO in the presence of BSA. (A)** RT-PCR of baNOS mRNA in different culture conditions. Left and middle panels compare the aerated and static conditions. Left and right panels show the effect of BSA (1 mg/ml). Aerated culture was grown in 2 ml of medium per well of a 6-well plate at 300 rpm at 37°C, 5% CO_2_ for 20 h. Static culture was grown in 10 ml of complete DMEM/F12 medium per well of a 6-well plate without shaking at 37°C, 5% CO_2_ for 20 h. 12.5 ng of bacterial RNA isolated with the TrizolMax kit (Invitrogen) were used per 50 μl reactions in 38 PCR cycles. No bands were detected in the negative controls without the RT step (not shown). **(B)** Cultures were growth in 5 ml of DMEM/F12 medium per well of a 12-well plate with shaking at 200 rpm at 37°C, 5% CO_2_ for 24 h. Cultures were supplemented with BSA (1 mg/ml) and/or NO-synthase inhibitor L-NAME (10 mM). Nitrite/nitrate concentration was measured with a colorimetric assay kit (Cayman Chemical). Error bars indicate 95% confidence intervals.

**Figure 2:**
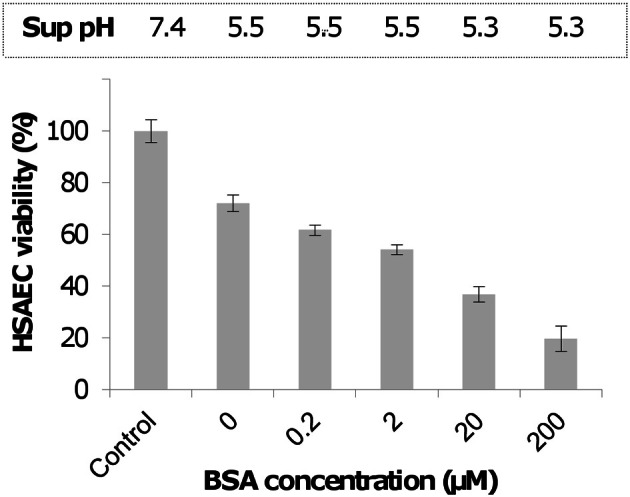
**Supplementation of complete DMEM/F12 culture medium with BSA increases the toxicity of Sups of dSterne cultures grown in microaerobic conditions for 24 h.** HSAECs were exposed to Sups for 2 h. Untreated cells served as control. OD_600_ of Sups was measured in 96-well plate reader (200 μl per well). Error bars indicate 95% confidence intervals.

The microaerobic cultures of the toxigenic (Sterne) and non-toxigenic (dSterne) strains upon their static growth on top of the HSAEC monolayers in the atmosphere of 5% CO_2_ demonstrate an acute cytotoxicity. The bacterial factors responsible for the toxicity are present in the bacterial culture supernatants (Sups) grown until late stationary phase (>20 h) (Popova et al., 2011). To elucidate contribution of baNOS to the toxicity of Sups we characterized the production of NO by this enzyme. We first demonstrated using RT-PCR that both strains expressed the baNOS gene in the stationary phase of growth (Figure 1A). Next, we analyzed Sups for accumulation of NO in the form of its end oxidation products, nitrite and nitrate. To avoid masking of the released nitrite/nitrate by the nitrate concentration in the culture medium such as CSFM (which contains *ca*. 300 μM nitrate), we used DMEM/F12 medium with low nitrate content (*ca.* 0.1 μM). In the aerated cultures of both strains the concentration of nitrite/nitrate did not exceeded a few μM, but strongly increased in the case when the medium was supplemented with BSA (Figure 1B). This increase was not accompanied by the increased transcription of baNOS (Figure 1A, left and right panels). The inhibitor of NOS (L-N^ω^-nitroarginine methyl ester, L-NAME) reduced the nitrite/nitrate content to the background level. These observations demonstrated that the accumulation of nitrite/nitrate resulted from the NO-generating activity of baNOS in the presence of BSA and suggested that the enhancing property of BSA might contribute to the toxicity of Sups. We tested the viability of HSAECs after incubation with Sups of bacterial cultures grown in the microaerobic conditions in the presence of different concentrations of BSA. Figure 2 shows a profound decrease of the HSAEC viability dependent on the concentration BSA in culture medium upon incubation with dSterne Sups. Similar results were obtained with the Sterne strain (not shown). The presence of BSA did not result in substantial changes of the optical densities of cultures (OD_600_ 0.30 ± 0.06 SD, *n* = 5). Therefore, the effect of BSA could not be attributed just to the larger number of bacteria. Instead, the increased acidity of Sups indicated changes in the production of acidic metabolic products (Figure 2).

### *B. anthracis* microaerobic cultures generate no-derived oxidants

**Figure 3:**
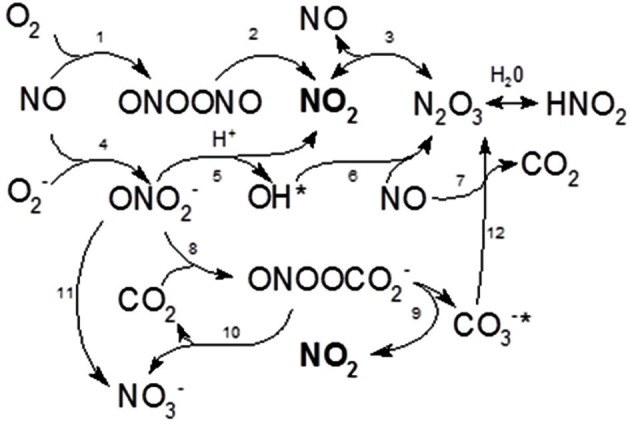
**Schematic of biologically relevant chemical reactions of NO with oxygen and superoxide radical summarizing results of Ford et al. (1993), Wink et al. (1993), Goldstein and Czapski (1995), Kharitonov et al. (1995), Goldstein et al. (1998), Espey et al. (2002a), Lancaster (2006), Lewis and Deen (1994), and Goldstein and Grabski (1995).** In the presence of oxygen, NO is autooxidized to NO_2_ which can further react with NO to form N_2_O_3_ (Reactions **1**–**3**). Reaction **1** is much slower in comparison with Reaction **4** which takes place in the presence of superoxide and results in the formation of peroxynitrite. The latter is unstable and decomposes with the formation of NO_2_ and hydroxyl radical which is able to quickly recombine with NO. Peroxynitrite can be rapidly consumed by CO_2_ to form the unstable nitrosoperoxocarbonate (ONOOCO^−^_2_) (reaction **8**) ultimately decomposing into NO_2_, nitrate and carbonate. As a result, NO_2_ accumulates as a major toxic species before its conversion to stable nitrite. The nitrate and nitrite can be metabolized for respiration in hypoxic conditions or biosynthesis. The stoichoimetric coefficients are not shown.

**Figure 4:**
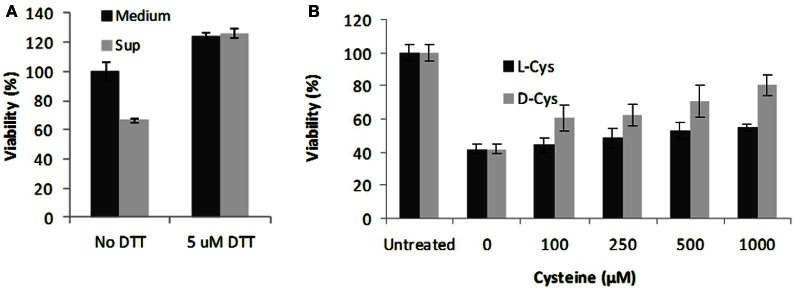
**Antioxidant treatment of Sups protects HSAECs.** The dSterne Sups grown in complete DMEM/F12 were incubated with 5 mM DTT for 30 min **(A)** or indicated concentrations of cysteine for 1 h **(B)**. HSAECs were exposed to the treated Sups for 2 h and the viability of cells was tested using Alamar Blue. In control experiments, addition of Cys had no effect on viability of untreated cells, and no cytoprotection was detected with other L - amino acids tested (Val, Leu, Ala) (not shown). Error bars indicate 95% confidence intervals.

To explain the enhancing effect of BSA on the toxicity of Sups we suggested that BSA might trap a volatile and highly diffusible NO as intermediate product(s) of chemical reactions with the protein side groups (Denicola et al., 1996b; Hakim et al., 1996; Pacher et al., 2007; Foster et al., 2009). It is also known that the BSA hydrophobic interior can reversibly absorb NO, thus decreasing its dissipation from solution and increasing the rate of NO autoxidation into intermediate species such as N_2_O_3_ or NO^·^_2_ (Rafikova et al., 2002) (Figure 3). In the presence of a sufficient amount of superoxide, which may originate in Sups during bacterial growth from various sources including the leakage of the respiratory chain (González-Flecha and Demple, 1995; Messner and Imlay, 1999, 2002; Dröse and Brandt, 2012), the trapped NO will be quickly converted to peroxynitrite and its decomposition products (Reaction **4** in Figure 3). The ROS/NOS could also be released from the Sup-exposed host cells (Popova et al., 2011). These reactive species in turn can rapidly oxidize a number of biological compounds, such as thiols, which can act as RNS/ROS scavengers (Pacher et al., 2007; Wouters et al., 2011). In agreement with these considerations, incubation of Sups with DTT or cysteine decreased the Sups' toxicity toward HSAECs (Figure 4).

To elucidate the nature of reactive species in Sups formed during bacterial growth we used the properties of various dyes to form characteristic products upon interaction with ROS and RNS. Amplex Red (10-Acetyl-3,7-dihydroxyphenoxazine, AR) is a sensitive dye typically used to detect hydrogen peroxide (H_2_O_2_) due to the formation of a highly fluorescent and colored product resorufin in the presence of horseradish peroxidase (HRP) (Zhou et al., 1997). This enzyme can also catalyze rapid oxidation of the dye by ONOO^−^ (formed in reaction of NO with superoxide, O^·−^_2_) in the presence or absence of carbon dioxide (Floris et al., 1993). However, distinguishing between peroxynitrite and H_2_O_2_-dependent mechanisms can be done based on the effect of inhibitors (e.g., L-NAME, superoxide dismutase, and catalase). The assay is quite specific regarding the nature of the oxidant and does not generate significant amounts of fluorescent products when exposed to 100 μM HOCl, xanthine/oxidase-generated O^·−^_2_, an anaerobic NO^·^ or H_2_O_2_ in the absence of HRP (Palazzolo-Ballance et al., 2007).

**Figure 5:**
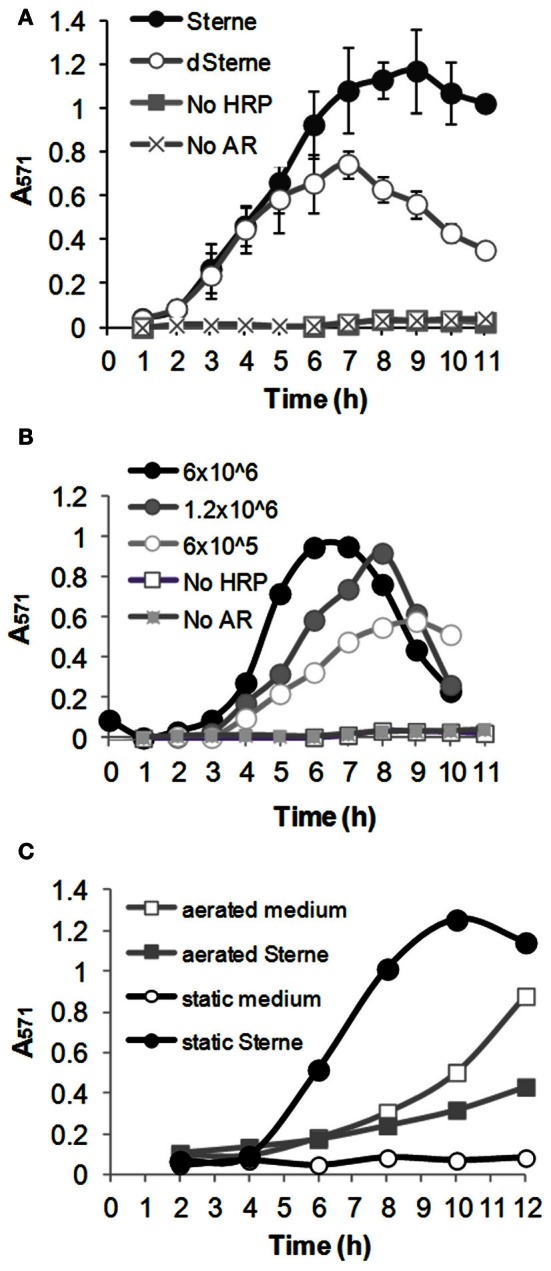
***B. anthracis* generates oxidizing species in microaerobic cultures.** Medium was seeded with spores, and cultures were grown at 37°C, 5% CO_2_ in the presence of 0.2 U/ml HRP and 100 μM Amplex Red (AR). Samples of bacterial suspensions were withdrawn at indicates times. Bacteria were pelleted for 5 min and absorbance of Sups was read at 571 nm. **(A)** Comparison of Sterne and dSterne strains. Cultures were seeded into 5 ml of CSFM per a well of a 12-well plate at 6.0 × 10^6^ spores/ml and grown without shaking. **(B)** Dose dependence of AR oxidation by dSterne strain seeded at the indicated number of spores/ml grown as in **(A)**. In comparison with **(A)**, bacterial suspensions in **(B)** were mixed 1:1 with 1 M Tris-HCl, pH 7.4, to correct for the possible effect of pH change on the absorbance of resorufin. **(C)** Comparison of static and aerated cultures for Sterne strain. Cultures were grown in 6-well plates seeded at 6.0 × 10^6^ spores/ml. For aeration the plates were shaken at 200 rpm. Error bars indicate standard deviations (*n* = 3).

To reliably detect transient reactive species we used a cumulative experimental setup in which AR and HRP were present in the medium during the whole period of bacterial growth. Aliquots of bacterial culture were withdrawn at certain time points, bacteria pelleted, and the Sups analyzed spectrophotometrically. The data in Figure 5 show that upon growth in static cultures both the toxigenic Sterne and the non-toxigenic dSterne strains were able to oxidize a colorless AR into a red-colored resorufin detected by absorbance in culture medium seeded with the indicated amount of spores. Accumulation of resorufin due to the release of oxidants took place in the spore dose-dependent manner and was followed by a gradual decline. In order to elucidate the mechanism behind the decline we first demonstrated that addition of H_2_O_2_ to Sups during the declination phase resulted in the appearance of color in accordance with the calibration curve obtained for H_2_O_2_. This indicated that the assay was able to respond to the additional oxidant and its components were not depleted (not shown). On the other hand, it is known that peroxidases can further oxidize resorufin into a colorless product (Towne et al., 2004). Therefore, we tentatively attributed this effect to the activity of HRP and limited our observations to the initial ascending parts of the curves. We found that Sterne strain was more productive than dSterne (Figure 5A), and control experiments without bacteria in static conditions with and without AR and HRP demonstrated only a low background level of oxidation. In contrast, aeration of the medium containing AR and HRP without bacteria caused a strong gradual oxidation of the dye which is known to be sensitive to air (Figure 5C). In these conditions, the growing Sterne bacteria did not oxidize AR and even reduced it below the background level (Figure 5C) while dSterne bacteria had smaller effect (not shown). This result reaffirmed that the decreased amount of available oxygen in static cultures served as a critical factor in controlling the generation of oxidizing species by *B. anthracis*.

### Effects of catalase and SOD are consistent with the transient formation of peroxynitrite

**Figure 6:**
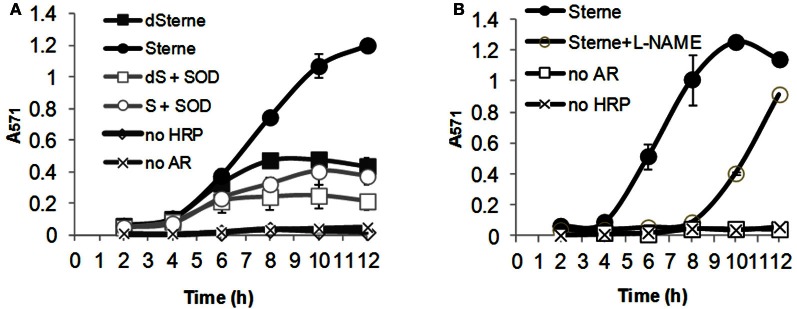
**SOD and L-NAME but not catalase inhibit oxidation of AR during bacterial growth in microaerobic conditions. (A)** Effect of SOD and catalase. In the presence of catalase (20 μg/ml) the response is indistinguishable from that of Sterne strain only. **(B)** Effect of L-NAME. Sups were prepared by growing bacteria in static conditions at 37°C, 5% CO_2_ in 24-well plate containing 1.5 ml of DMEM/F-12 medium per well **(A)** or 12-well plate containing 5 ml of DMEM/F12 medium per well **(B)**. Spores were seeded to a final concentration of 6 × 10^6^/ml. SOD, HRP, AR, and L-NAME final concentrations were 10 U/ml, 0.2 U/ml, 0.1 mM, and 10 mM, correspondingly. Before spectrophotometric measurements, bacteria were pelleted by centrifugation and Sups were diluted 1:1 with 10 × PBS, pH 7.4, to exclude the effect of pH change on the absorbance of resorufin. Error bars indicate standard deviations (*n* = 3).

To distinguish between the release of H_2_O_2_ and other oxidants like peroxynitrite, the test was supplemented with catalase (up to 20 μg/ml). The Sterne strain in the presence of catalase showed the intensity of resorufin indistinguishable from the bacteria without catalase (Figure 6A) thus excluding H_2_O_2_ as the reactive species generated by *B. anthracis*. On the other hand, it was reported that HRP can use peroxynitrite as substrate in the catalase-insensitive process (Floris et al., 1993). Experiments with the addition of 10 U/ml of *E. coli* Mn-superoxide dismutase (Mn-SOD) strongly inhibited oxidation. This effect, however, cannot be interpreted as evidence of the direct interaction of superoxide radical with SOD because the dismutation product, H_2_O_2_, would readily react with AR thus maintaining its oxidation at the same level (Zielonka et al., 2012). It is also considered unlikely that SOD would be able to compete effectively with the extremely fast conversion of superoxide into peroxynitrite in the presence of NO (Pacher et al., 2007). Therefore, we favor the mechanism in which Mn-SOD is involved in a direct catalytic inactivation of peroxynitrite (Quijano et al., 2001; Surmeli et al., 2010).

### Activity of baNOS is involved in the production of oxidizing and toxic species in Sups

**Figure 7:**
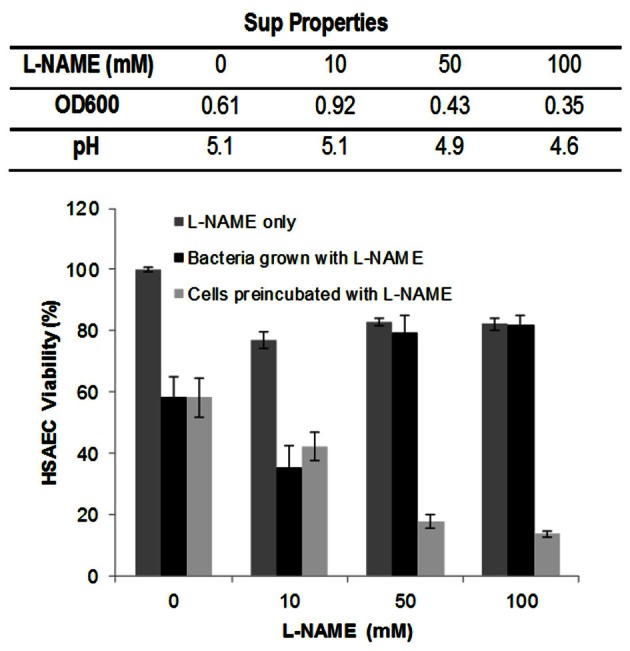
**Inhibition of baNOS with L-NAME decreases toxicity of Sups to HSAECs.** Static cultures of dSterne strain were grown in complete DMEM/F12 as described in Materials and Methods with or without the indicated concentrations of L-NAME. The table shows the ODs and pHs of 24-h cultures. The Sups were added for 2 h to HSAECs pretreated or not with the indicated concentrations of L-NAME for 1 h, and the viability of the cells was assayed with Alamar Blue. Controls included the culture medium with L-NAME without bacteria. All tested samples were titrated with HCl to the pH 5.1 of Sups without L-NAME. Error bars indicate 95% confidence intervals.

One might expect that the formation of peroxynitrite or other NO-derived RNS should depend on the activity of baNOS as a major source of NO. Indeed, addition of L-NAME to the culture medium strongly reduced the oxidation of AR (Figure 6B). We also tested if the reduced oxidation in the presence of baNOS correlated with the reduced toxicity of Sups to HSAECs. The culture media were supplemented with different concentrations of L-NAME and inoculated with equal amounts of dSterne spores. The static cultures were grown for 24 h. It was found that inhibition of baNOS affected the bacterial growth and increased the generation of acidic products (Figure 7). The mechanism of this effect has not been reported and the nature of the metabolic changes is currently unknown. To compare Sups grown in the presence and absence of L-NAME in the viability test we wanted to exclude the unspecific effect of pH differences between them which would mask the contribution of other Sup components. However, the reduced pH is important for the toxicity of Sups which become non-toxic when titrated to pH > 6 (Popova et al., 2011). Therefore all samples were titrated with a small volume of NaOH to the acidity of Sups without L-NAME (pH 5.1). In these conditions the initial assayed concentration of L-NAME (10 mM) slightly decreased the viability of untreated and treated HSAECs; however, the viability relative to the corresponding controls without and with L-NAME remained statistically unchanged (mean ± 95% CI were 58 ± 7% vs. 48 ± 9%, correspondingly). Higher concentrations of L-NAME caused a strong protection up to the level of control cells treated with L-NAME only (Figure 7).

One caveat of the above experiments was that during the viability test the L-NAME present in Sups might potentially inhibit the host cell NOS along with the baNOS. To clarify this possibility, HSAECs were pre-incubated with L-NAME for 1 h, the inhibitor was removed, and the cells were exposed to the Sups of cultures grown without L-NAME. In contrast to the effect of L-NAME on bacteria, the viability of HSAECs was decreased considerably, indicating the protective effect of the host NOS against the toxic substances of Sups.

### Tyrosine nitration and oxidation of dihydrorhodamine 123 (DHR) confirm generation of peroxynitrite

**Figure 8:**
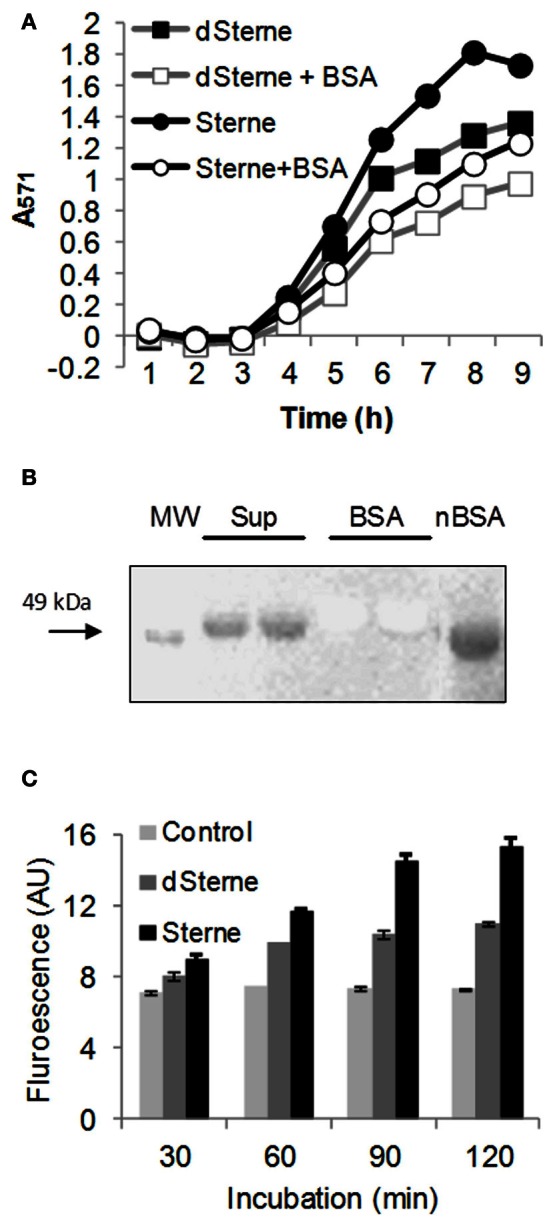
**Bacterial growth in microaerobic conditions generates oxidizing species with the properties of PN. (A)** BSA inhibits the oxidation of AR catalyzed by HRP in dSterne cultures grown as in Figure 6 in 12-well plates with the addition of 1 mg/ml of BSA in DMEM/F12 medium. **(B)** Western blot of dSterne Sups, unmodified BSA, and nitrated BSA (nBSA) using anti-nitrotyrosine antibodies. Sups were prepared by inoculation of dSterne spores (final concentration of 6 × 10^6^ spores/ml) into DMEM/F12 with 1 mg/ml of BSA. After 24 h, bacteria were removed by centrifugation and gel electrophoresis was performed followed by membrane transfer. Anti-rabbit HRP-linked secondary antibody was used. Sup and BSA lanes are identical duplicates. **(C)** Fluorescence of DHR indicates the release of peroxynitrite in *B. anthracis* cultures. Bacteria grown statically for 18 h in complete DMEM/F12 medium were pelleted, washed with PBS, resuspended in PBS containing DHR, and incubated at 37°C, 5% CO_2_ for the indicate periods of time for detection of fluorescence at 500/540 nm. Error bars indicate 95% confidence intervals.

Current methodologies for detection of peroxynitrite are based on the reactions of radical species formed from its decomposition. The formation of nitrotyrosine (TyrNO_2_) from tyrosine residues of proteins is commonly considered as evidence of the transient peroxynitrite presence resulting in the release of intracellular ^·^NO_2_ (Pacher et al., 2007; Ferrer-Sueta and Radi, 2009). The results of the AR/HRP test showed that the addition of BSA to the culture medium resulted in a partial consumption of the RNS formed in the Sups (Figure 8A) indicating that BSA could be a convenient substrate to detect possible reactions of Tyr nitration. Therefore we analyzed the chemical modification of BSA in the dSterne Sup by western blotting with anti-TyrNO_2_ antibodies. We detected a band corresponding to the nitrated BSA in the Sups (Figure 8B) but not in the original medium. As a positive control we used a partial nitration of BSA by an acidified sodium nitrite which accompanies the main reaction of protein S-nitrosylation [Stamler et al. (1992) and our data presented below].

DHR serves as a sensitive fluorogenic probe for peroxynitrite-derived RNS (NO_2_, hydroxyl radical OH^·^, or carbonate radical CO^·−^_3_) (Wrona et al., 2005; Zielonka et al., 2012). Initial experiments with DHR added to the bacterial cultures growing in complete DMEM/F12 demonstrated a strong quenching the DHR signal; therefore, a modified protocol was used. Bacteria grown for a certain period of time were pelleted, washed with PBS, finally resuspended in PBS containing DHR and incubated at 37°C, 5% CO_2_ for the appearance of fluorescence. Figure 8C shows that bacteria-generated species were able to convert DHR into fluorescent product indicating the transient formation of peroxynitrite, which is unlikely to accumulate in Sups due to its short lifetime.

### BSA cysteine residues are not in involved in the generation of toxicity

Cys-34 is one of the BSA residues highly susceptible to various chemical modifications by ROS and RNS, because it is located in the hydrophobic cavity formed by the protein tertiary structure and is not involved in the formation of disulfide bridges with other Cys residues of the protein (Christodoulou et al., 1995; Rafikova et al., 2002). Serum albumin isolated from plasma contains only about 65% of reduced thiol (King, 1961; Era et al., 1995). The oxidized portion of Cys-34 was thought to be in a mixed disulfide form, mostly with cysteine, cysteinyl-glycine (Cys-Gly) (a degradation product of glutathione), and glutathione (GSH) to a lesser extent (King, 1961; Yasuhara and Nokihara, 1998). It has been previously suggested that the thiol content of serum albumin is important for the transport and stabilization of NO through the formation of intermediate nitrosothiol derivatives (Foster et al., 2009).

**Figure 9:**
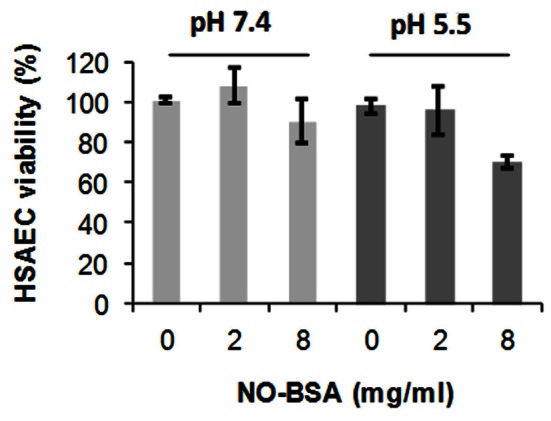
**Nitrosylated BSA (NO-BSA) is not acutely toxic.** HSAECS were incubated with NO-BSA at indicated concentrations and pH for 2 h in DMEM/F12 medium, and the viability of HSAECs was tested using Alamar Blue. Error bars indicate 95% confidence intervals.

Our results of AR test presented above showed that *B. anthracis* microaerobic cultures represent an oxidizing environment. Therefore we expected that the free thiol groups of Cys-34 would be oxidized or otherwise chemically modified during the bacterial growth and wanted to test if such modifications would contribute to the toxicity of Sups. First, we tested the viability of HSAECs after incubation of the BSA with acidified nitrite, which results mainly in the S-nitrosylation of Cys-34 (Stamler et al., 1992). There was only a marginal effect of nitrosylated BSA on cell viability detected at the concentration of 8 mg/ml at the pH 7.4 corresponding to the culture medium or pH 5.3–5.5 corresponding to Sups (Figure 9). This result excluded S-nitrosylation from the candidate BSA modifications resulting in the generation of toxic species in the Sups and suggested a protective contribution of free thiols due to partial consumption of NO in this reaction.

**Figure 10:**
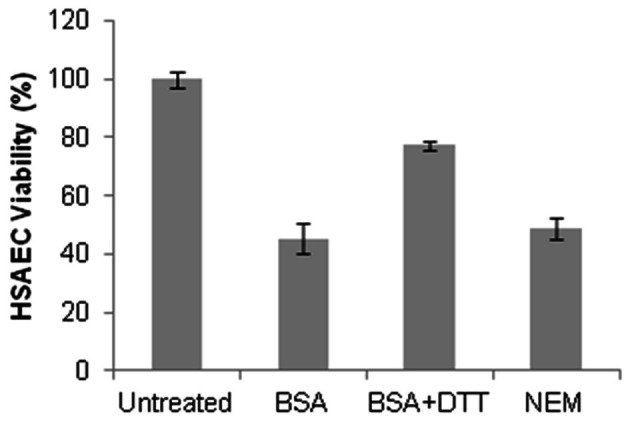
**Blocking of free SH groups of BSA does not reduce the toxicity of dSterne Sup grown in the presence of BSA.** The protein was dissolved in 10 ml of PBS, treated with 5 mM DTT for 30 min, and then dialyzed against two changes of 1 l of PBS for 24 h. The reduced BSA was then modified by NEM (125 μM) for 1 h at 37°C and dialyzed against two changes of 1 l of PBS overnight. The modified and mock-treated BSA was used to supplement the DMEM/F12 medium at 2 mg/ml. Bacterial cultures were grown for 24 h and Sups were tested for toxicity using HSAECs and the Alamar Blue viability assay. Error bars indicate 95% confidence intervals.

To test the above suggestion, we increased the free-thiol content of BSA by reducing it with 5 mM DTT for 30 min followed by an extensive dialysis against PBS. The SH content of BSA determined using a thiol-specific Ellman's reagent (5,5′-dithiobis-2-nitrobenzoic acid, DTNB) was increased two-fold after the DTT treatment (to about 90% of total Cys-34), but the Sup prepared using this BSA was found to be less toxic than the one with the untreated BSA (Figure 10). However, this effect cannot be attributed solely to the chemical reactivity of SH groups. The cultures supplemented with the reduced BSA had almost a three-fold reduction in bacterial growth and a decreased acidity in comparison with the non-reduced BSA (data not shown) indicating substantial metabolic changes in the reduced environment.

Finally, we chose to block Cys-34 residues by N-ethylmaleimide (NEM) and compare the toxicity of Sups supplemented with the modified and unmodified BSA. After the NEM treatment the free-thiol content of the BSA dropped to <5%, compared with the amount found in the unmodified BSA. The cultures with the modified and unmodified BSA showed no substantial differences between each other in the growth of bacteria, final pH, and the toxicity of Sups (Figure 10). Overall, we concluded that free SH groups of BSA were not required for the accumulation of toxicity during bacterial growth and even partially antagonized it similar to the effects of DTT and cysteine after incubation with Sups (Figure 4).

### Mass spectroscopy (MS) analysis of BSA modification products in Sups confirms the presence of no-derived chemical modifications

For the MS analysis we used the Sterne Sup grown in CSFM. This medium contains 1 mg/ml of BSA. The Sups were treated with trypsin, and the peptide fragments with S-nitrosylatedCys and nitrated Tyr were identified by the LC-MS/MS. Controls included the untreated BSA as well as the BSA modified with sodium nitrite in acidic conditions. Table 1 shows that the nitrite-modified BSA digest contained a single S-nitrosylated, Cys-34-containing peptide along with several Tyr-nitrated ones. The static Sterne culture demonstrated selective S-nitrosylation of Cys-34 and nitration of Tyr-30. No nitration was found in the aerated culture, which might reflect a reduced amount of peroxynitrite formed in comparison with the hypoxic conditions of static culture in agreement with the AR test results (Figure 5C). However, S-nitrosylation of Cys-34 was still detectable, indicating that peroxynitrite was not required for this reaction.

**Table 1:** Chemical modifications of BSA identified in Sups of *B. anthracis* Sterne cultures grown in CSFM^*^.

**Sample**	**Peptides with S-nitrosylation**	**Peptides with Tyr-nitration**
**Untreated BSA**	None	None
**Nitrite-treated BSA (positive control)**	G^21^LVLIAFSQY^30^LQQC#PFDEHVK	RHPEY@AVSVLLR
		HPYFY@APELLYYANK
		HPYFYAPELLY@YANK
		HPYFYAPELLYY@ANK
		RHPY@FY@APELLYYANK
		LGEY@GFQNALIVR
		DAFLGSFLY@EYSR
		DAFLGSFLYEY@SR
**Static culture**	GLVLIAFSQYLQQC#PFDEHVK	GLVLIAFSQY@LQQC#PFDEHVK
**Aerated culture**	GLVLIAFSQYLQQC#PFDEHVK	None

* CSFM contains 1 mg/ml of BSA.

# Indicates S-nitrosylation.

@ Indicates Tyr nitration.

### Oxidation of Sups with permanganate abrogates toxicity

**Figure 11:**
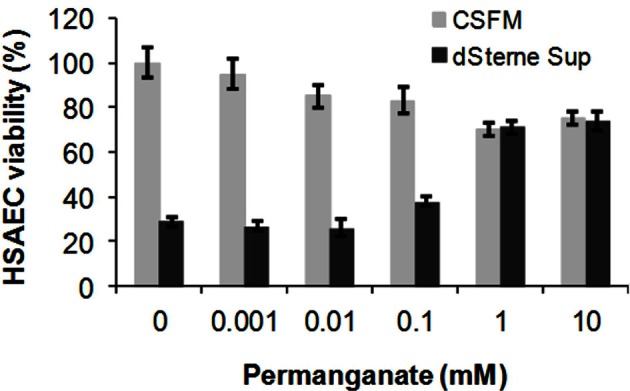
**Permanganate treatment reduces the toxicity of dSterne Sup.** The indicated concentrations of KMnO_4_ were added for 1 h to the Sup grown in CSFM for 24 h, and toxicity of the treated Sup was tested after incubation with HSAECs for 2 h. Error bars indicate 95% confidence intervals.

Peroxynitrite is a strong oxidant [E′_o_ 1.4 v for ONOO^−^/NO^−^_3_ (Zakharova et al., 2007)]. However, the many of its biological effects can be due to the reactions of its decomposition product, NO_2_ (Pacher et al., 2007) [E′_o_ 0.8 v for NO_2_/NO^−^_3_ (Standard electrode potential data)]. We suggested that an oxidant stronger than peroxynitrite and NO_2_ will be able to abrogate Sup toxicity by converting these species to a non-toxic nitrate. Permanganate is the oxidant capable of eliminating both peroxynitrite and NO_2_ in close to neutral conditions [E′_o_ 1.7 v for MnO^−^_4_/MnO_2_ (Standard electrode potential data)]. It has been previously used for a chemical titration of the peroxynitrite solutions (Sturzbecher et al., 2007). We incubated dSterne Sups with a range of potassium permanganate concentrations for 1 h and found a strong protection of HSAECs from Sup toxicity at >0.1 mM KMnO_4_ (Figure 11). The permanganate itself cased only a relatively small decrease in cell viability.

## Discussion

In this study we further elucidated the recently discovered mechanism of *B. anthracis* toxicity toward the host cells which requires a hypoxic environment for bacterial growth and an accumulation of acidic metabolic products (Popova et al., 2011). We show that *B. anthracis* was able to generate micromolar concentrations of NO in the process sensitive to the baNOS inhibitor, L-NAME (Figure 2). Unexpectedly, we found that accumulation of the nitrite/nitrate (as products tracing the presence of NO) required a supplementation of the culture medium with BSA. We suggested that this effect reflects the property of BSA to absorb NO into the hydrophobic interior of its globule and thus facilitate the autoxidation of NO in the presence of oxygen (Rafikova et al., 2002). Estimates show that the acceleration effect due to the increased concentrations of reactants can reach >10^4^ times (Rafikova et al., 2002). Although the fine details of the oxidation mechanism are still disputed, all authors agree that NO_2_ is an intermediate Ford et al., 1993; Lewis and Deen, 1994; Goldstein and Czapski, 1995; Kharitonov et al., 1995; Lancaster, 2006) which can be formed in several reactions (Figure 3). Further interaction of NO with NO_2_ can give rise to N_2_O_3_. However, the latter species can be present only at very low concentrations compared to NO_2_ (Espey et al., 2002a), which is known to be an active oxidant of a number of biological species including sulfhydryl compounds (Wink et al., 1993; Goldstein and Czapski, 1995). Therefore the process of BSA interaction with NO in the presence of oxygen will likely involve chemical reaction of NO or NO_2_ with the BSA side chain groups, such as the free thiol group of Cys-34. According to (Rafikova et al., 2002), about 5% of the absorbed NO becomes converted to a relatively stable S-nitrosoalbumin. Literature data (Ishima et al., 2009) and our experiments (Figure 9) show that these BSA derivatives are not acutely toxic and are expected to act as NO_2_ sinks, removing a portion of oxidant from the system. Similar reactions can explain the effect of free Cys or DTT on the Sup activity (Figure 4). However, the generation of the NO-related toxicity by bacteria overwhelms the effect of free BSA thiols (and possibly other reactive protein groups).

Regardless of the reactive intermediates involved, nitrosation *via* NO autoxidation may be the dominant route when relatively high rates of NO formation occur in the presence of oxygen without significant O^−^_2_ generation (Wink et al., 1993; Liu et al., 1998; Espey et al., 2001). In contrast, the formation of peroxynitrite may chiefly prevail under conditions where O^−^_2_ is present, but not in great excess of NO (Pacher et al., 2007; Zielonka et al., 2012) (Reaction **4**). The rapid reaction between O^−^_2_ and NO (Koppenol et al., 1992; Goldstein and Czapski, 1995; Kissner et al., 1997) has prompted numerous investigators to focus on peroxynitrite and the roles it may play in oxidation and nitration of susceptible molecules (Radi et al., 2001). The protonated peroxynitrite anion can decompose leading to secondary formation of other oxidants, chiefly NO_2_ and hydroxyl radical (OH^·^, reaction **5**) (Koppenol et al., 1992; Kissner et al., 1997; Goldstein et al., 1998). While NO_2_ is capable of permeating cells (Liu et al., 1998; Espey et al., 2002b), it is likely that the highly reactive OH^·^ molecule would not have sufficient lifetime for diffusion into the cell and will recombine with NO to form a stable nitric acid (reaction **6**)(Espey et al., 2002a).

An important facet to consider in the biochemistry of NO and O^·−^_2_ is the influence of CO_2_ on this system. Several groups (Denicola et al., 1996a; Lymar et al., 1996; Jourd'heuil et al., 1999; Romero et al., 1999; Khairutdinov et al., 2000; Zhang et al., 2001) have demonstrated that ONOO^−^ can be rapidly consumed by CO_2_ to form the CO_2_ adduct nitrosoperoxocarbonate (ONOOCO^−^_2_) (reaction **8**). This reaction pathway would circumvent the putative homolysis of ONOOH to NO_2_ (Reaction **5**) and ^·^OH in favor of ONOOCO^−^_2_ decomposition into NO_2_ and carbonate radical (CO^−^_3_, Reaction **9**). The CO_2_ reaction pathway may result in an enhanced level of NO_2_ formation relative to that produced by alternate routes of peroxynitrite catabolism. The literature data argue against ONOO^−^, ONOOH or ONOOCO^−^_2_ as significant cell permeable species relative to NO_2_ (Lymar et al., 1996; Khairutdinov et al., 2000). The commonality of NO_2_ as a putative product of the ONOOH or ONOOCO^−^_2_ decomposition pathways in combination with the ability of NO_2_ to diffuse into cells and mediate oxidation (Khairutdinov et al., 2000) suggest it as one of key toxic agents in Sups.

Experiments with AR confirmed that *B. anthracis* grown in microaerobic cultures generated oxidizing species. Based on the accumulation of resorufin in the presence of catalase the properties of this species were distinct from H_2_O_2_. In addition, the inhibiting effect of SOD indicated that this species was not the superoxide, because the SOD-catalyzed dismutation of the latter would give rise to H_2_O_2_ still capable of the AR oxidation. We also found that the oxidation of AR was inhibited by L-NAME and therefore concluded that the major reactive species originated from NO (which itself does not oxidize AR). In contrast to the microaerobic conditions, bacteria in aerated cultures did not generate oxidants and even decreased the background level of AR oxidation observed in culture medium without bacteria. Overall, the results are completely consistent with the formation of peroxynitrite in fast reaction **4** effectively competing with reaction **1** under microaerobic conditions. We did not investigate the origin of the superoxide in *B. anthracis* cultures. Similar to other bacteria, it may consist in the respiratory chain leakage or the activity of metabolic enzymes such as fumarate reductase (Messner and Imlay, 2002).

The formation of peroxynitrite provides an explanation for the increased fluorescence of DHR in response to bacterial products. DHR has been most widely used to measure intracellular oxidants. This probe does not directly react with H_2_O_2_ or peroxynitrite, but becomes oxidized via radical mechanism involving OH^·^, NO_2_, or CO^·−^_3_ in the presence of carbon dioxide (reactions **5, 8, 9**)(Wrona et al., 2005).

In the case peroxynitrite and NO_2_ are the major reactive species participating in the oxidation of AR, one should expect that their interaction with BSA would result in the formation of nitrated Tyr residues. Indeed, such a modification was detected using a specific antibody (Figure 8) as well as the MS analysis (Table 1) in static but not in aerobic conditions, consistent with the absence of bacteria-produced oxidizing species in aerated cultures tested with AR (Figure 5).

Finally, we carried out experiments with L-NAME added to the medium of bacterial cultures to demonstrate that inhibition of baNOS resulted in the reduced toxicity of Sups (Figure 7) correlating with the capacity of L-NAME to decrease the formation of AR-oxidizing species. The effect of L-NAME was accompanied by the reduced bacterial growth, raising a possibility that it might, at least in part, contribute to the decreased toxicity of Sups. However, it seems to be more likely that the decreased bacterial numbers, as well as the decreased Sup toxicity, in the presence of L-NAME are directly caused by the inhibition of baNOS playing regulatory and cytoprotective roles in several bacterial species including the *Bacilli* (Crane et al., 2010). We plan to resolve this question in our future research.

We showed that effective protection of HSAECs could be accomplished in reaction with permanganate as a strong oxidant more potent than peroxynitrite or NO_2_. This result suggests that the oxidizing agents might be considered for elimination of the toxic RNS generated by *B. anthracis*. While oxidation of NO_2_ can result in a relatively inert nitrate, a reduction of NO_2_ using antioxidants has a potential to recover a chemically reactive nitrite or biologically active NO.

The mechanism we describe was not previously identified for bacterial pathogens capable of producing NO either through the activity of NOS or denitrification. However, very similar cytotoxic effects supporting our observations were reported in the case of neural PC12 cells exposed to peroxynitrite generated during oxidation of 3-morpholinisydnonimide (SIN-1) (Konishi et al., 2009; Shirai et al., 2012). The exposure of cells to the culture medium obtained after complete SIN-1 decomposition demonstrates almost the same level of cytotoxicity as fresh SIN-1. Although the reactive species were not identified, their properties closely mirror our observations. The cytotoxicity is dependent on the presence of serum and could be abolished by thiols. The presence of SOD but not catalase during the SIN-1 decomposition prevents the formation of cytotoxic substances. The authors concluded that the formation of toxicants is a result of the simultaneous formation of NO and superoxide. In agreement with our considerations regarding the mechanism of peroxynitrite reactivity, it was found that CO_2_ plays a critical role in the cytotoxicity of the SIN-1 decomposition products (Shirai et al., 2012). Surprisingly, the authors misinterpreted the effect of BSA when they found that addition of fresh serum (or BSA) antagonizes the cytotoxicity. As we show, BSA consumes a portion of reactive species in chemical reactions with its side groups and therefore needs to be chemically “saturated” in order to display a maximal toxic effect.

In summary, we present novel data regarding the property of baNOS to participate in the formation of cytotoxic substances through generation of NO which gives rise to the transient formation of reactive species with the properties of peroxynitrite in the conditions of reduced oxygen availability. This product is known to quickly decompose with the accumulation of stable but chemically reactive NO_2_. Our data are consistent with the notion of NO_2_ as the major toxic species in the Sups. The role of BSA may consist in the trapping of NO and NO_2_ in the hydrophobic interior of the protein globule accompanied by chemical reactions of the BSA side chains. This mechanism is expected to function along with the toxic effect ALO and SA discovered in our previous studies. The experiments are in progress to elucidate a possible synergism between NO, ALO, and SA as pathogenic factors of *B. anthracis*.

### Conflict of interest statement

The authors declare that the research was conducted in the absence of any commercial or financial relationships that could be construed as a potential conflict of interest.

## References

[B1] Belik J. Stevens D. Pan J. McIntyre B. A. S. Kantores C. Ivanovska J. (2010). Pulmonary vascular and cardiac effects of peroxynitrite decomposition in newborn rats. Free Rad. Biol. Med. 49, 1306–1314 10.1016/j.freeradbiomed.2010.07.021 20688155

[B2] Bradburne C. Chung M.-C. Zong Q. Schlauch K. Liu D. Popova T. (2008). Transcriptional and apoptotic responses of THP-1 cells to challenge with toxigenic, and non-toxigenic *Bacillus anthracis*. BMC Immunol. 9:67 10.1186/1471-2172-9-67 19014542 PMC2613145

[B3] Bueno E. Mesa S. Bedmar E. J. Richardson D. J. Delgado M. J. (2012). Bacterial adaptation of respiration from oxic to microoxic and anoxic conditions: redox control. Antioxid. Redox Signal. 16, 819–852 10.1089/ars.2011.4051 22098259 PMC3283443

[B4] Chand H. S. Drysdale M. Lovchik J. Koehler T. M. Lipscomb M. F. Lyons C. R. (2009). Discriminating virulence mechanisms among *Bacillus anthracis* strains by using a murine subcutaneous infection model. Infect. Immun. 77, 429–435 10.1128/IAI.00647-08 18981254 PMC2612256

[B5] Christodoulou J. Sadler P. J. Tucker A. (1995). 1H NMR of albumin in human blood plasma: drug binding and redox reactions at Cys34. FEBS Lett. 376, 1–5 10.1016/0014-5793(95)01231-2 8521951

[B6] Chung M.-C. Narayanan A. Popova T. G. Kashanchi F. Bailey C. L. Popov S. G. (2013). *Bacillus anthracis*-derived nitric oxide induces protein S-nitrosylation contributing to macrophage death. Biochem. Biophys. Res. Commun. 430, 125–130 10.1016/j.bbrc.2012.11.042 23178574

[B7] Cote C. K. DiMezzo T. L. Banks D. J. France B. Bradley K. A. Welkos S. L. (2008). Early interactions between fully virulent *Bacillus anthracis* and macrophages that influence the balance between spore clearance and development of a lethal infection. Microbes Infect. 10, 613–619 10.1016/j.micinf.2008.02.006 18467145

[B8] Crane B. R. Sudhamsu J. Patel B. A. (2010). Bacterial nitric oxide synthases. Annu. Rev. Biochem. 79, 445–470 10.1146/annurev-biochem-062608-103436 20370423

[B9] Denicola A. Freeman B. A. Trujillo M. Radi R. (1996a). Peroxynitrite reaction with carbon dioxide/bicarbonate: kinetics and influence on peroxynitrite-mediated oxidations. Arch. Biochem. Biophys. 333, 49–58 10.1006/abbi.1996.0363 8806753

[B10] Denicola A. Souza J. M. Radi R. Lissi E. (1996b). Nitric oxide diffusion in membranes determined by fluorescence quenching. Arch. Biochem. Biophys. 328, 208–212 10.1006/abbi.1996.0162 8638932

[B11] Dixon T. C. Fadl A. A. Koehler T. M. Swanson J. A. Hanna P. C. (2000). Early *Bacillus anthracis*-macrophage interactions: intracellular survival survival and escape. Cell. Microbiol. 2, 453–463 10.1046/j.1462-5822.2000.00067.x 11207600

[B12] Dröse S. Brandt U. (2012). Molecular mechanisms of superoxide production by the mitochondrial respiratory chain. Adv. Exp. Med. Biol. 748, 145–169 10.1007/978-1-4614-3573-0_6 22729857

[B13] Era S. Kuwata K. Imai H. Nakamura K. Hayashi T. Sogami M. (1995). Age-related change in redox state of human serum albumin. Biochim. Biophys. Acta 1247, 12–16 7873580 10.1016/0167-4838(94)00166-e

[B14] Espey M. G. Miranda K. M. Thomas D. D. Wink D. A. (2001). Distinction between nitrosating mechanisms within human cells and aqueous solution. J. Biol. Chem. 276, 30085–30091 10.1074/jbc.M101723200 11404354

[B15] Espey M. G. Thomas D. D. Miranda K. M. Wink D. A. (2002a). Focusing of nitric oxide mediated nitrosation and oxidative nitrosylation as a consequence of reaction with superoxide. Proc. Natl. Acad. Sci. U.S.A. 99, 11127–11132 10.1073/pnas.152157599 12177414 PMC123221

[B16] Espey M. G. Xavier S. Thomas D. D. Miranda K. M. Wink D. A. (2002b). Direct real-time evaluation of nitration with green fluorescent protein in solution and within human cells reveals the impact of nitrogen dioxide vs. peroxynitrite mechanisms. Proc. Natl. Acad. Sci. U.S.A. 99, 3481–3486 10.1073/pnas.062604199 11904413 PMC122549

[B17] Ferrer-Sueta G. Radi R. (2009). Chemical biology of peroxynitrite: kinetics, diffusion, and radicals. ACS Chem. Biol. 4, 161–177 10.1021/cb800279q 19267456

[B18] Floris R. Piersma S. R. Yang G. Jones P. Wever R. (1993). Interaction of myeloperoxidase with peroxynitrite. A comparison with lactoperoxidase, horseradish peroxidase and catalase. Eur. J. Biochem. 215, 767–775 10.1111/j.1432-1033.1993.tb18091.x 8394811

[B19] Ford P. C. Wink D. A. Stanbury D. M. (1993). Autoxidation kinetics of aqueous nitric oxide. FEBS Lett. 326, 1–3 10.1016/0014-5793(93)81748-O 8325356

[B20] Foster M. W. Hess D. T. Stamler J. S. (2009). Protein S-nitrosylation in health and disease: a current perspective. Trends Mol. Med. 15, 391–404 10.1016/j.molmed.2009.06.007 19726230 PMC3106339

[B21] Goldstein S. Czapski G. (1995). The reaction of NO. with O^−^_2_ and HO_2_: a pulse radiolysis study. Free Rad. Biol. Med. 19, 505–510 10.1016/0891-5849(95)00034-U 7590401

[B22] Goldstein S. Grabski G. (1995). Kinetics of nitric oxide autoxidation in aqueous solution in the absence and presence of various reductants: the nature of the oxidizing intermediates. J. Am. Chem. Soc. 117, 12078–12084

[B23] Goldstein S. Czapski G. Lind J. Merenyi G. (1998). Mechanism of decomposition of peroxynitric ion (O(2)NOO(-)): evidence for the formation of O(2)(^*^-) and (^*^)NO(2) radicals. Inorg. Chem. 37, 3943–3947 10.1021/ic980051l 11670507

[B24] González-Flecha B. Demple B. (1995). Metabolic sources of hydrogen peroxide in aerobically growing *Escherichia coli*. J. Biol. Chem. 270, 13681–13687 10.1074/jbc.270.23.13681 7775420

[B25] Guichard A. Nizet V. Bier E. (2012). New insights into the biological effects of anthrax toxins: linking cellular to organismal responses. Microbes Infect. 14, 97–118 10.1016/j.micinf.2011.08.016 21930233 PMC3743078

[B26] Guidi-Rontani C. (2002). The alveolar macrophage: the Trojan horse of *Bacillus anthracis*. Trends Microbiol. 10, 405–409 10.1016/S0966-842X(02)02422-8 12217505

[B27] Habib S. Ali A. (2011). Biochemistry of nitric oxide. Ind. J. Cli. Biochem. 26, 3–17 10.1007/s12291-011-0108-4 22211007 PMC3068772

[B28] Hakim T. S. Sugimori K. Camporesi E. M. Anderson G. (1996). Half-life of nitric oxide in aqueous solutions with and without haemoglobin. Physiol. Meas. 17, 267–277 10.1088/0967-3334/17/4/004 8953625

[B29] Heninger S. Drysdale M. Lovchik J. Hutt J. Lipscomb M. F. Koehler T. M. (2006). Toxin-deficient mutants of *Bacillus anthracis* are lethal in a murine model for pulmonary anthrax. Infect. Immun. 74, 6067–6074 10.1128/IAI.00719-06 16923785 PMC1695493

[B30] Inglesby T. V. O'Toole T. Henderson D. A. Bartlett J. G. Ascher M. S. Eitzen E. (2002). Anthrax as a biological weapon, 2002: updated recommendations for management. JAMA 287, 2236–2252 10.1001/jama.287.17.2236 11980524

[B31] Inglesby T. V. (2002). Anthrax as a biological weapon, 2002: updated recommendations for management. JAMA 287, 2236–2252 10.1001/jama.287.17.2236 11980524

[B32] Ishima Y. Kragh-Hansen U. Maruyama T. Otagiri M. (2009). Albumin as a nitric oxide-traffic protein: characterization, biochemistry and possible future therapeutic applications. Drug Metab. Pharmacokinet. 24, 308–317 10.2133/dmpk.24.308 19745558

[B33] Jourd'heuil D. Miranda K. M. Kim S. M. Espey M. G. Vodovotz Y. Laroux S. (1999). The oxidative and nitrosative chemistry of the nitric oxide/superoxide reaction in the presence of bicarbonate. Arch. Biochem. Biophys. 365, 92–100 10.1006/abbi.1999.1143 10222043

[B34] Khairutdinov R. F. Coddington J. W. Hurst J. K. (2000). Permeation of phospholipid membranes by peroxynitrite. Biochemistry 39, 14238–14249 10.1021/bi001270x 11087373

[B35] Kharitonov V. G. Sundquist A. R. Sharma V. S. (1995). Kinetics of nitrosation of thiols by nitric oxide in the presence of oxygen. J. Biol. Chem. 270, 28158–28164 10.1074/jbc.270.47.28158 7499306

[B36] King T. P. (1961). On the sulfhydryl group of human plasma albumin. J. Biol. Chem. 236, PC5 13756115

[B37] Kissner R. Nauser T. Bugnon P. Lye P. G. Koppenol W. H. (1997). Formation and properties of peroxynitrite as studied by laser flash photolysis, high-pressure stopped-flow technique, and pulse radiolysis. Chem. Res. Toxicol. 10, 1285–1292 10.1021/tx970160x 9403183

[B38] Konishi K. Watanabe N. Arai T. (2009). SIN-1 cytotoxicity to PC12 cells is mediated by thiol-sensitive short-lived substances generated through SIN-1 decomposition in culture medium. Nitric Oxide 20, 270–278 10.1016/j.niox.2009.02.001 19232545

[B39] Koppenol W. H. Moreno J. J. Pryor W. A. Ischiropoulos H. Beckman J. S. (1992). Peroxynitrite, a cloaked oxidant formed by nitric oxide and superoxide. Chem. Res. Toxicol. 5, 834–842 1336991 10.1021/tx00030a017

[B40] Kraft B. Strous M. Tegetmeyer H. E. (2011). Microbial nitrate respiration – Genes, enzymes and environmental distribution. J. Biotechnol. 155, 104–117 10.1016/j.jbiotec.2010.12.025 21219945

[B41] Lancaster J. R. (2006). Nitroxidative, nitrosative, and nitrative stress: kinetic predictions of reactive nitrogen species chemistry under biological conditions. Chem. Res. Toxicol. 19, 1160–1174 10.1021/tx060061w 16978020

[B42] Levy H. Weiss S. Altboum Z. Schlomovitz J. Glinert I. Sittner A. (2012a). Differential contribution of *Bacillus anthracis* toxins to pathogenicity in two animal models. Infect. Immun. 80, 2623–2631 10.1128/IAI.00244-12 22585968 PMC3434592

[B43] Levy H. Weiss S. Altboum Z. Schlomovitz J. Rothschild N. Glinert I. (2012b). The effect of deletion of the edema factor on *Bacillus anthracis* pathogenicity in guinea pigs and rabbits. Microb. Pathog. 52, 55–60 10.1016/j.micpath.2011.10.002 22020310

[B44] Lewis R. S. Deen W. M. (1994). Kinetics of the reaction of nitric oxide with oxygen in aqueous solutions. Chem. Res. Toxicol. 7, 568–574 7981422 10.1021/tx00040a013

[B45] Liu X. Miller M. J. Joshi M. S. Thomas D. D. Lancaster J. R. (1998). Accelerated reaction of nitric oxide with O2 within the hydrophobic interior of biological membranes. Proc. Natl. Acad. Sci. U.S.A. 95, 2175–2179 9482858 10.1073/pnas.95.5.2175 PMC19287

[B46] Lovchik J. A. Drysdale M. Koehler T. M. Hutt J. A. Lyons C. R. (2012). Expression of either lethal toxin or edema toxin by *Bacillus anthracis* is sufficient for virulence in a rabbit model of inhalational anthrax. Infect. Immun. 80, 2414–2425 10.1128/IAI.06340-11 22526673 PMC3416453

[B47] Lymar S. V. Jiang Q. Hurst J. K. (1996). Mechanism of carbon dioxide-catalyzed oxidation of tyrosine by peroxynitrite. Biochemistry 35, 7855–7861 10.1021/bi960331h 8672486

[B48] Messner K. R. Imlay J. A. (1999). The identification of primary sites of superoxide and hydrogen peroxide formation in the aerobic respiratory chain and sulfite reductase complex of Escherichia coli. J. Biol. Chem. 274, 10119–10128 10.1074/jbc.274.15.10119 10187794

[B49] Messner K. R. Imlay J. A. (2002). Mechanism of superoxide and hydrogen peroxide formation by fumarate reductase, succinate dehydrogenase, and aspartate oxidase. J. Biol. Chem. 277, 42563–42571 10.1074/jbc.M204958200 12200425

[B50] Moayeri M. Leppla S. H. (2009). Cellular and systemic effects of anthrax lethal toxin and edema toxin. Mol. Aspects Med. 30, 439–455 10.1016/j.mam.2009.07.003 19638283 PMC2784088

[B51] Pacher P. Beckman J. S. Liaudet L. (2007). Nitric oxide and peroxynitrite in health and disease. Physiol. Rev. 87, 315–424 10.1152/physrev.00029.2006 17237348 PMC2248324

[B52] Palazzolo-Ballance A. M. Suquet C. Hurst J. K. (2007). Pathways for intracellular generation of oxidants and tyrosine nitration by a macrophage cell line. Biochemistry 46, 7536–7548 10.1021/bi700123s 17530864 PMC2584613

[B53] Popov S. G. Villasmil R. Bernardi J. Grene E. Cardwell J. Wu A. (2002). Lethal toxin of *Bacillus anthracis* causes apoptosis of macrophages. Biochem. Biophys. Res. Commun. 293, 349–355 10.1016/S0006-291X(02)00227-9 12054607

[B54] Popova T. G. Millis B. Chung M.-C. Bailey C. Popov S. G. (2011). Anthrolysin O and fermentation products mediate the toxicity of *Bacillus anthracis* to lung epithelial cells under microaerobic conditions. FEMS Immunol. Med. Microbiol. 61, 15–27 10.1111/j.1574-695X.2010.00740.x 20946354 PMC3040846

[B55] Quijano C. Hernandez-Saavedra D. Castro L. McCord J. M. Freeman B. A. Radi R. (2001). Reaction of peroxynitrite with Mn-superoxide dismutase. Role of the metal center in decomposition kinetics and nitration. J. Biol. Chem. 276, 11631–11638 10.1074/jbc.M009429200 11152462

[B56] Quinlan C. L. Orr A. L. Perevoshchikova I. V. Treberg J. R. Ackrell B. A. Brand M. D. (2012). Mitochondrial complex II can generate reactive oxygen species at high rates in both the forward and reverse reactions. J. Biol. Chem. 287, 27255–27264 10.1074/jbc.M112.374629 22689576 PMC3411067

[B57] Radi R. Peluffo G. Alvarez M. N. Naviliat M. Cayota A. (2001). Unraveling peroxynitrite formation in biological systems. Free Rad. Biol. Med. 30, 463–488 10.1016/S0891-5849(00)00373-7 11182518

[B58] Rafikova O. Rafikov R. Nudler E. (2002). Catalysis of S-nitrosothiols formation by serum albumin: the mechanism and implication in vascular control. Proc. Natl. Acad. Sci. U.S.A. 99, 5913–5918 10.1073/pnas.092048999 11983891 PMC122876

[B59] Romero N. Denicola A. Souza J. M. Radi R. (1999). Diffusion of peroxynitrite in the presence of carbon dioxide. Arch. Biochem. Biophys. 368, 23–30 10.1006/abbi.1999.1272 10415107

[B60] Russell B. H. Liu Q. Jenkins S. A. Tuvim M. J. Dickey B. F. Xu Y. (2008). *In vivo* demonstration and quantification of intracellular *Bacillus anthracis* in lung epithelial cells. Infect. Immun. 76, 3975–3983 10.1128/IAI.00282-08 18625737 PMC2519418

[B61] Shatalin K. Gusarov I. Avetissova E. Shatalina Y. McQuade L. E. Lippard S. J. (2008). *Bacillus anthracis*-derived nitric oxide is essential for pathogen virulence and survival in macrophages. Proc. Natl. Acad. Sci. U.S.A. 105, 1009–1013 10.1073/pnas.0710950105 18215992 PMC2242674

[B62] Shirai K. Okada T. Konishi K. Murata H. Akashi S. Sugawara F. (2012). Bicarbonate plays a critical role in the generation of cytotoxicity during SIN-1 decomposition in culture medium. Oxid. Med. Cell. Longev. 2012:326731 10.1155/2012/326731 22848780 PMC3400428

[B63] Stamler J. S. Simon D. I. Osborne J. A. Mullins M. E. Jaraki O. Michel T. (1992). S-nitrosylation of proteins with nitric oxide: synthesis and characterization of biologically active compounds. Proc. Natl. Acad. Sci. U.S.A. 89, 444–448 1346070 10.1073/pnas.89.1.444 PMC48254

[B64] Standard electrode potential (data page) – Wikipedia, the Free Encyclopedia. Available online at: http://en.wikipedia.org/wiki/Standard_electrode_potential_(data_page) (Accessed December 25, 2012).

[B65] Sturzbecher M. Kissner R. Nauser T. Koppenol W. H. (2007). Homolysis of the peroxynitrite anion detected with permanganate. Inorg. Chem. 46, 10655–10658 10.1021/ic7013978 18001115

[B66] Sudhamsu J. Crane B. R. (2009). Bacterial nitric oxide synthases: what are they good for? Trends Microbiol. 17, 212–218 10.1016/j.tim.2009.02.003 19375324

[B67] Surmeli N. B. Litterman N. K. Miller A.-F. Groves J. T. (2010). Peroxynitrite mediates active site tyrosine nitration in manganese superoxide dismutase. Evidence of a role for the carbonate radical anion. J. Am. Chem. Soc. 132, 17174–17185 10.1021/ja105684w 21080654 PMC3050995

[B68] Towne V. Will M. Oswald B. Zhao Q. (2004). Complexities in horseradish peroxidase-catalyzed oxidation of dihydroxyphenoxazine derivatives: appropriate ranges for pH values and hydrogen peroxide concentrations in quantitative analysis. Anal. Biochem. 334, 290–296 10.1016/j.ab.2004.07.037 15494136

[B69] Valez V. Cassina A. Batinic-Haberle I. Kalyanaraman B. Ferrer-Sueta G. Radi R. (2012). Peroxynitrite formation in nitric oxide-exposed submitochondrial particles: detection, oxidative damage and catalytic removal by Mn-porphyrins. Arch. Biochem. Biophys. 529, 45–54 10.1016/j.abb.2012.10.012 23142682 PMC3534903

[B70] Wink D. A. Darbyshire J. F. Nims R. W. Saavedra J. E. Ford P. C. (1993). Reactions of the bioregulatory agent nitric oxide in oxygenated aqueous media: determination of the kinetics for oxidation and nitrosation by intermediates generated in the NO/O2 reaction. Chem. Res. Toxicol. 6, 23–27 8448345 10.1021/tx00031a003

[B71] Wouters M. A. Iismaa S. Fan S. W. Haworth N. L. (2011). Thiol-based redox signalling: rust never sleeps. Int. J. Biochem. Cell Biol. 43, 1079–1085 10.1016/j.biocel.2011.04.002 21513814

[B72] Wrona M. Patel K. Wardman P. (2005). Reactivity of 2',7'-dichlorodihydrofluorescein and dihydrorhodamine 123 and their oxidized forms toward carbonate, nitrogen dioxide, and hydroxyl radicals. Free Rad. Biol. Med. 38, 262–270 10.1016/j.freeradbiomed.2004.10.022 15607909

[B73] Yasuhara T. Nokihara K. (1998). Quantitative determination of biological sulfhydryl groups by postcolumn derivatization and elucidation of microheterogeneity of serum albumins. Anal. Chem. 70, 3505–3509 9726170 10.1021/ac9802630

[B74] Zakharova E. A. Yurmazova T. A. Nazarov B. F. Wildgoose G. G. Richard G. C. (2007). The voltammetric determination of peroxynitrite at a mercury film electrode. New J. Chem. 31, 394–400

[B75] Zhang H. Joseph J. Feix J. Hogg N. Kalyanaraman B. (2001). Nitration and oxidation of a hydrophobic tyrosine probe by peroxynitrite in membranes: comparison with nitration and oxidation of tyrosine by peroxynitrite in aqueous solution. Biochemistry 40, 7675–7686 10.1021/bi002958c 11412121

[B76] Zhou M. Diwu Z. Panchuk-Voloshina N. Haugland R. P. (1997). A stable nonfluorescent derivative of resorufin for the fluorometric determination of trace hydrogen peroxide: applications in detecting the activity of phagocyte NADPH oxidase and other oxidases. Anal. Biochem. 253, 162–168 10.1006/abio.1997.2391 9367498

[B77] Zielonka J. Zielonka M. Sikora A. Adamus J. Joseph J. Hardy M. (2012). Global profiling of reactive oxygen and nitrogen species in biological systems: high-throughput real-time analyses. J. Biol. Chem. 287, 2984–2995 10.1074/jbc.M111.309062 22139901 PMC3270955

